# Transformation of Engineered Copper Oxide Nanoparticles in Surface Waters

**DOI:** 10.3390/jox14040078

**Published:** 2024-10-06

**Authors:** Patrice Turcotte, Christian Gagnon

**Affiliations:** Environment & Climate Change Canada, Water Science & Technology, 105 McGill St., Montréal, QC H2Y 2E7, Canada

**Keywords:** transformation, nanomaterials, metals, natural waters, particle size

## Abstract

Copper oxide nanoparticles (CuO-NPs) are widely used for their catalytic properties, conductive capacity, and innovations in the fields of superconductors, alloys, and solar energy sensors. To better understand the impact of water chemistry on the stability of CuO nanoparticles, a series of measurements were carried out on nanoparticles suspended in pure water, natural water, and water enriched with natural organic matter fulvic acid (FA). ICP-MS characterization in single-particle mode (SP-ICP-MS) was performed to determine the stability or transformation of nanoparticles in contrasting water conditions. We first observed that particle sedimentation was very fast in pure Milli-Q water. The addition of FA favored the dissolution of CuO-NPs with an increase in the dissolved copper concentration, for both Milli-Q water and natural water. The presence of FA also reduced the size of CuO-NPs (i.e., less aggregation) measured in natural water. By comparing signals of single particles, FA decreased nanoparticle numbers as well, confirming the increase in dissolution of CuO-NPs over time. The transformation products of CuO-NPs are important in the ecological context since the uptake and toxicity of parent nanoparticles differ from those of the chemical species in solution. Further considerations are needed on the fate of released NPs to better assess their exposure pathways to aquatic organisms and potential environmental risks.

## 1. Introduction

Metal nanoparticles from nanotechnology production have different properties than those of metal bulk due to their small sizes and surface chemistry. Copper oxide nanoparticles (CuO-NPs) are widely used for their catalytic properties and conductive capacity for innovations in the fields of superconductors, alloys, and solar energy sensors [1]. Cu nanoparticles are also applied in various commercial products, mainly due to their antimicrobial properties. After use, released NPs can pose an ecotoxicological risk to exposed aquatic organisms [2]. While manufacturer properties of various NPs are known and often documented by industry, these properties are determined for stock solutions sold and do not include environmental matrix effects. Little of this information is applicable for the evaluation of their transformation after use and release to complex mixtures, such as wastewater and surface waters containing natural organic matter.

Because some dissolution of metal particles is likely to occur, it is important to distinguish the effects of nanoparticulate forms from dissolved metal ions (e.g., [3]). To assess the environmental impacts of nanoparticles (NPs), their transformation and fate must be assessed in various aquatic environments, including natural waters and wastewater discharges [4,5]. Some initial characteristics of pristine NPs, such as size and surface coating, can be altered or modified in the receiving medium and constitute an analytical challenge. It is expected that the organic matter present in the environment, including natural organic substances and wastewater, will contribute to a natural coating on the NP [6,7], which must be considered (i.e., the influence on transformation processes) for a more accurate assessment of their environmental risk. Liu et al. [8] showed that these coated forms aggregate in different aqueous matrices and that their hydrodynamic particle volumes are much larger than the pristine nanoparticles. Also, they noted that there was increased dissolution relative to the pristine NPs. However, their studies do not make it possible to measure whether there are size intermediate species between the nanoparticle aggregate and the fragmented and dissolved forms thereof.

Higher uptake from Cu-based NPs than an equivalent ionic Cu exposure was reported for aquatic organisms, such as D. magna and zebrafish, suggesting that NP forms are bioavailable to organisms [3]. Different toxicity mechanisms of action were also reported for nanoparticulate and ionic forms. For example, exposure to CuO-NPs induced lower SOD activity in mussel digestive glands compared to those exposed to Cu ions, in addition to the observed induction of metallothionein [9]. Exposure of CuO-NPs to juvenile trout revealed that NPs could produce effects in fish gills without showing Cu accumulation [10]. Similar results were reported for bivalves exposed to CuO-NPs, where accumulation of Cu was not observed in mussels, but specific effects, compared to ionic forms, were observed related to inflammation and protein damage [11]. Ecotoxicological studies, thus, highlighted the importance of considering all transformed forms in toxicity investigations.

The study of the transformation and persistence of these nanoparticles in the aquatic environment requires the use of analytical techniques to distinguish the transformed forms of metals, assessing whether they are in particulate, nanoparticle, colloidal, or dissolved form following exposure to the aquatic environment. To measure the size distribution and concentration of nanoparticles in environmental samples, the “single-particle” mode with an inductively-coupled plasma-mass spectrometer (SP-ICP-MS) is suitable for such size distribution analyses at environmental concentrations [12,13,14]. This instrumental analysis allowed us to measure the size and assess some transformations of nanoparticulate metals, such as silver and copper oxide, in aqueous media at environmentally relevant concentrations [15].

The environmental risk assessment of CuO-NPs in waters should include studies of their molecular stability in different environments [16]. An understanding of their transformation is needed for more realistic assessments of their exposure routes, which are influenced or controlled by size distribution [17]. The environmental effects of nanoparticulate forms are expected to differ from transformed NPs or dissolved forms following transformation processes, such as precipitation, aggregation, and dissociation.

Environmental monitoring of CuO-NPs in aquatic ecosystems or in laboratory experiments requires a sensitive analytical approach that will allow the differentiation of CuO-NPs from dissolved Cu at low environmental concentrations. ICP-MS is commonly used for the analysis of total Cu at low levels. Such sensitive ICP-MS technique allows single-particle sample-inclusion mode to distinguish NPs from transformation products, such as metal ions. This analytical approach can also be used to distinguish between dissolved forms and CuO-NPs with the single-particle approach (SP-ICP-MS) at environmentally relevant concentrations.

This specific method, working at environmentally relevant copper concentrations (ng/L), was shown to be effective for the detection of metal NPs in natural waters and wastewaters [18]. This method recently upgraded with the selection of specific targeted atomic mass for the successful detection of AgNPs, which considers that the isobaric interference of natural particles on the detection of synthetic NPs [15,19] could be applied to the detection of metal oxide nanoparticles.

Experiments of the transformation kinetics of CuO nanoparticles under controlled conditions in different types of water were carried out for a better evaluation of factors controlling their fate in the aquatic environment. Pure Milli-Q water, which contains no minerals or organic matter, was utilized for comparison purposes.

## 2. Materials and Methods

### 2.1. Materials

Copper oxide (CuO) nanoparticles (CAS RN 1317-38-0) were purchased from US Research Nanomaterials Inc. #US7566 as a 20% suspended nanoparticles stock solution with a size range of 25–55 nm. Auclair et al. [11] obtained the hydrodynamic diameters via DLS in Milli-Q water, 79 ± 10 and 80 ± 10 nm after 1 h and 48 h, respectively. In surface water (pH 8.1 ± 0.5, conductivity 297 ± 30 µScm^−1^, and TOC 3.1 ± 1 mg/L), they were 100 ± 36 and 133 ± 18 nm after 1 h and 48 h, respectively. The size differences between the certificate and Auclair et al. [11] suggested that the CuO-NPs would tend to aggregate in working solutions.

### 2.2. Analysis of Copper by ICP-MS (ICAP-RQ)

The ICP-MS analysis of copper (^65^Cu) was reported to show some polyatomic isobaric interferences [20]. Analysis of Cu by ICP-MS using the isotope 65 showed isobaric interferences with Mg associated with Ar (^40^Ar^25^Mg). These were eliminated with the use of the KED (Kinetic Energy Discrimination) interference correction mode available on the ICP-MS (ICAP-RQ from Thermo Fisher Scientific), with a mixture of He (93%)/H_2_ (7%) as a collision gas. The technique is based on the collision of interferences with the gas, and the analyte also collides. Since the polyatomic isobaric interference was larger than the analyte, the collision frequency was greater for the interference, and it was eliminated [18]. On the other hand, the use of this mode led to a loss of analyte sensitivity. The average ICP-MS sensitivity was near 15,000 counts per second (CPS) for 1.0 µg Cu/L. For the SP-ICP-MS technique, this translated to a higher size detection limit compared to an element such as silver, which is much more sensitive (170000 CPS). While the size detection limit for AgNP was close to 15 nm, that for CuO-NPs was near 45 nm under our experimental conditions.

### 2.3. NP Characterization by Single-Particle ICP

**Figure 1:**
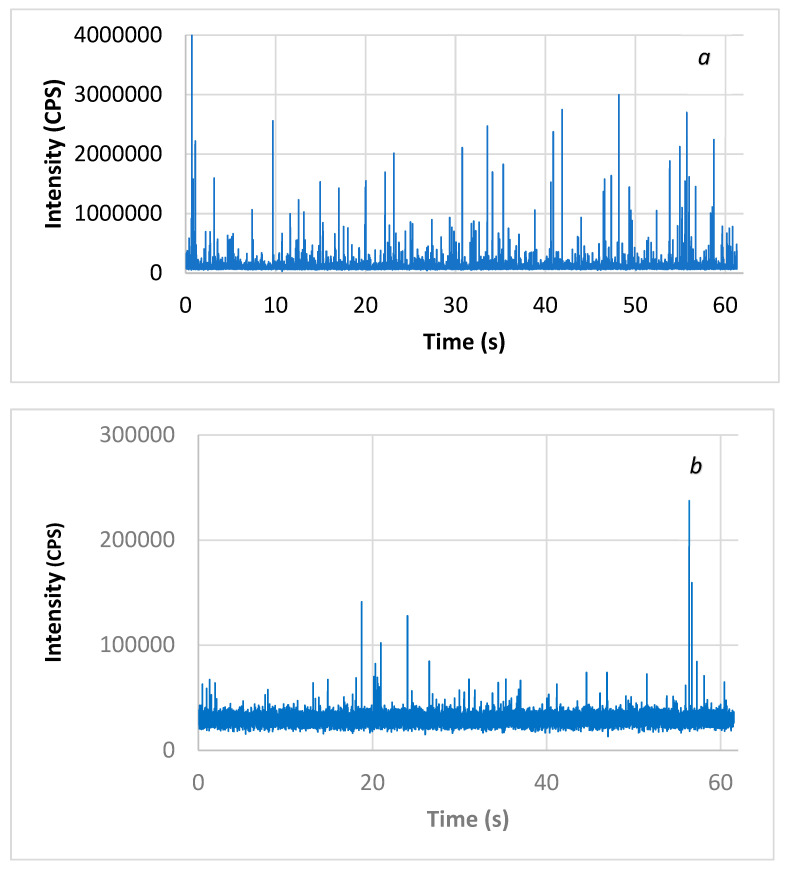
Raw signal of CuO-NPs in (**a**) Milli-Q pure water diluted 1:25 and (**b**) surface water analyzed by the SP-ICP-MS technique.

CuO-NPs’ size distributions in waters were evaluated by ICP-MS with the single-particle technique (SP-ICP-MS). The analytical technique consisted of collecting a series of transiting measurements on the order of a millisecond for at least 1 min in ICP-MS. As observed results, the dissolved phase distributed uniformly in the solution generated a constant baseline signal, while the passage of individual particles of CuO in the ICP plasma was characterized by an intense peak signal for a short period of time (0.5 ms). Figure 1 shows the raw signal from the analysis of CuO-NPs in Milli-Q pure water and surface water. Within the resulting signal, one can easily distinguish the presence of a pulse associated with the passage of CuO-NPs in the ICP plasma. The intensity of this signal was proportional to the size of the particle. A short reading time, on the order of a millisecond (ms), allowed this specific signal to be collected.

The mathematical processing of all the signals collected made it possible to separate and quantify the particle and ion species [21]. Such data processing also allowed characterizing particle spherical size distributions (size classes). However, this technique did not make it possible to characterize the shape of the particle nor to identify whether the Cu particle was part of a heterogeneous aggregate.

Data processing was performed with NP-Quant software (version 1) from Thermo Fisher Scientific. To maximize the intensity signal, an acquisition time per event (dw) of 2.5 ms, for a total time of 60 s, was chosen. The transport rate that relates the CuO-NP count detected by ICP-MS to that in the sample was evaluated with certified AgNP and was 10%. The total Cu concentration in solution was determined by an ICP instrument calibrated with Cu ion standards (SCP Science). Thus, the total concentration was calculated from average intensities (CPS) of a sample and the ICP-MS sensitivity (CPS/1.0 µ Cu/L). ICP-MS sensitivity, CPS for 1 µ Cu/L, and the NP-CuO CPS were also used to calculate the mass concentrations in ppt (ng/L) of CuO-NPs. With the method’s entirety, the number of CuO-NPs, their particle size, and the total Cu concentration in solution were determined.

### 2.4. Transformation Experiments

CuO-NP solutions of 6.25 µg total Cu/L were prepared in pure Milli-Q (MQ) water with or without 7 mg/L of added fulvic acids (FA; Suwannee River, Georgia, U.S.A.), and surface water (SW) with or without added FA (7 mg/L). The surface water (St. Lawrence River, Canada) used for these experiments had a conductivity of nearly 280 µS/cm, a dissolved organic carbon concentration of nearly 3 mg/L, and a slightly basic pH of 8.1. The pH of MQ water was 5.0, and with the added 7 ppm of fulvic acid, this shifted to 4.2 and the conductivity was 9.6 µS/cm.

Regarding particle size distribution, results were grouped into five size classes (nm): 48 to 55, 56 to 76, 77 to 137, 138 to 171, and >171 nm, to highlight the types of transformation products. For each size class, the number of particles detected per analysis cycle of 60 s (particles/cycle) and the Cu concentration, expressed as ng of Cu/L, were both presented. Concentrations (ng/L) of total copper and total dissolved Cu/small colloids were also provided. The dissolved Cu/small colloid concentration was defined as anything below 48 nm. Before each of these analyses by the SP-ICP-MS mode, the solutions were diluted by a factor of 25 with MQ water for a final concentration of 250 ng/L. Before the dilution, the 6.25 µg/L solution was not shaken for the analysis of the suspension only, not the precipitate. One of the goals of the experiments was to evaluate the sedimentation of CuO-NPs. Also, the experiments sought to determine if part of the precipitated CuO-NP forms could be remobilized since aggregated forms can be expected to transform and remobilize NPs in suspension over time.

## 3. Results and Discussion

**Figure 2:**
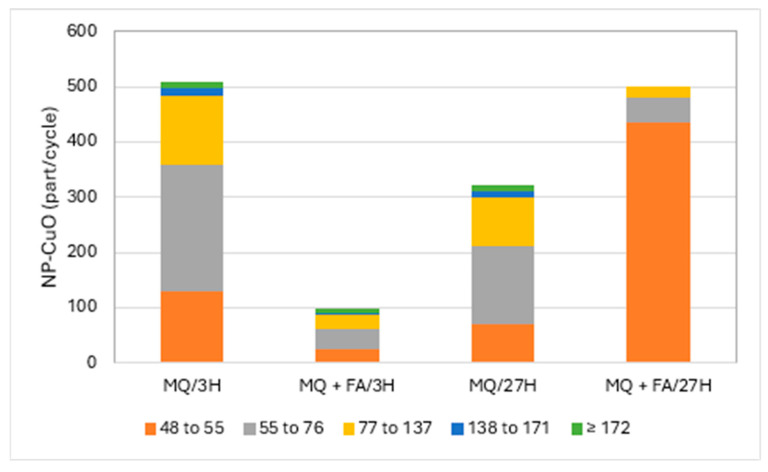
CuO-NPs’ (part/cycle) size (nm) distribution in Milli-Q water (MQ) and with added fulvic acids (FA) after 3 h and 27 h.

**Figure 3:**
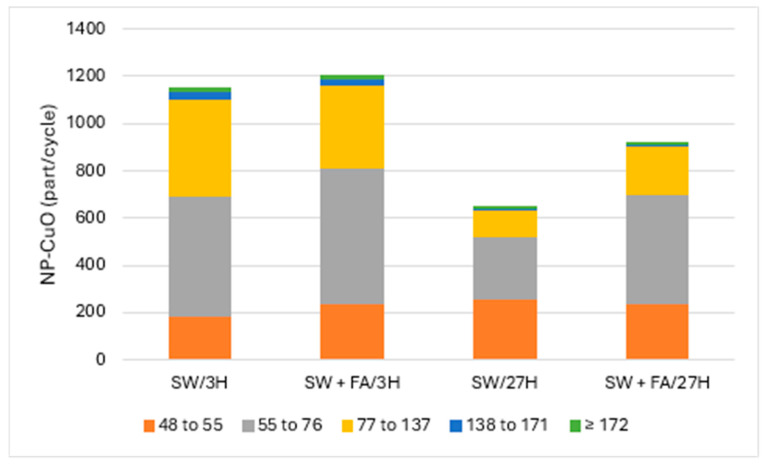
CuO-NPs’ (part/cycle) size (nm) distribution in surface water (SW) and with added fulvic acids (FA) after 3 h and 27 h.

**Figure 4:**
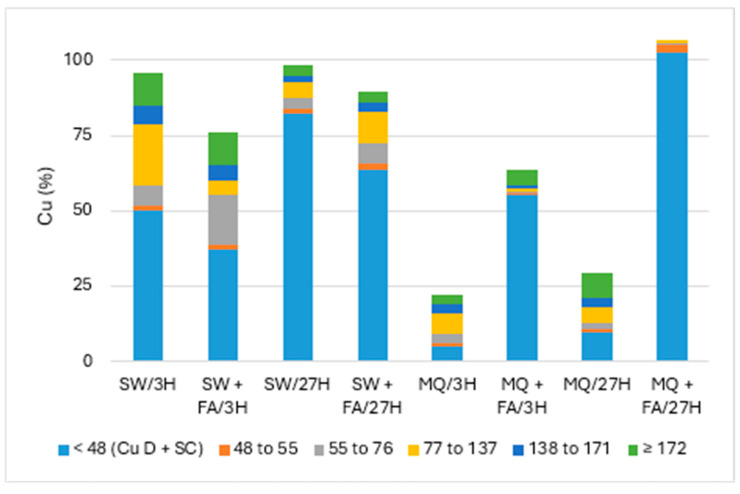
Proportion of Cu (nm) remaining in solution: CuO-NPs’ dispersion in pure (MQ) and surface (SW) waters and with added fulvic acid (FA) after 3 h and 27 h.

Figure 2 and Figure 3 present the particle numbers detected for each of the defined size classes, as well as concentrations of total Cu and dissolved Cu plus the small colloids fraction (<48 nm) in solutions analyzed after 3 h and 27 h (i.e., 1 day later). The proportions expressed in % relative to the nominal concentration of 250 ng Cu/L (concentration without sedimentation) were calculated for total Cu, dissolved Cu plus small colloids (D + SC), and nanometric Cu (NP; Figure 4).

### 3.1. Characterization of CuO-NPs in Pure Milli-Q Water

Results demonstrated that CuO-NPs tended to precipitate in Milli-Q (MQ) water. The solution of CuO-NPs in MQ water after 3 h showed a total concentration of 57.5 ng/L, while the prepared solution should show 250 ng of Cu/L as an initial concentration. Thus, only 23% of total Cu was recovered in the remaining suspension solution. The concentration of dissolved copper/small colloids was 11.9 ng/L, which represents 21% of the Cu remaining in solution and only 5% in comparison with the theoretical concentration of 250 ng/L. Such results could also be associated with the transformation of CuO-NPs. We also took for granted that the degraded CuO-NPs fraction must remain in solution and that particulate forms are precipitating. The rest of the Cu in suspension was nanoparticulate (CuO-NPs), i.e., 18% compared to the initial concentration (250 ng/L). CuO-NPs were found in all the defined size classes, though mainly in the classes 55 to 76 and 77 to 137 nm. These sizes were comparable to those obtained by Auclair et al. [11] using a DLS technique. The same solution, after 27 h, showed less precipitation of CuO-NPs, where 35% of the initial Cu was present, with the proportion of dissolved Cu/small colloids increasing from 5 to 10%. Here, part of the CuO-NPs degraded into dissolved/small colloids within 27 h. The nanometric Cu fraction in solution, for both periods, remained relatively constant at ~20%. After 27 h, CuO-NPs, expressed in number of particles, were still present in all the defined particle size classes, but at lower particle numbers for the four last fractions (>55 nm), with an increase in the fraction of 55–77 nm. These results suggest degradation of large NPs or NP aggregate degradation into smaller NPs. Figure 2 presents, as an example, the number of particles detected for each of the defined size classes in pure MQ water solutions with and without fulvic acid (FA) for the analysis in both periods.

Solutions of CuO-NPs in MQ water to which fulvic acids were added showed a different behavior. The total Cu concentration after 3 h was 159.5 ng/L, or 64% of the prepared nominal concentration, compared to 23% for the pure MQ water solution without fulvic acids. The differences were considerable in the proportion of the dissolved fraction/small colloids, which rose to 55% in the presence of FA, which was accompanied by a decrease in the proportion of nanometric CuO-NPs. The presence of FA promoted Cu suspension in solution and the degradation of CuO-NPs. This degradation of CuO-NPs with FA added to MQ water was also confirmed by comparing the particle detection numbers, which were much lower for all the classes (Figure 2). The recovery rate of total Cu at 27 h compared to the nominal solution was almost 100%, with a large majority in the dissolved fraction/small colloids. The proportion of nanometric Cu, however, compared to the initial concentration, was only 3%. This low proportion resulted from lower detection of large particles. Thus, no particle detection was observed in the upper two particle size classes, while the two classes preceding them remained relatively constant. The difference was mainly marked by an increase in the number of particle detections in the 48–55 nm class as a result of the partial degradation of CuO-NPs into smaller particles by fulvic acid or the resuspension of precipitated CuO-NPs by stabilizing FA.

A trend that emerged from this study was a weak degradation of the CuO-NP moiety in pure Milli-Q water on a daily basis. (Figure 2). On the other hand, the degradation was much more pronounced with the addition of FA. Struyk et al. [22] showed that humic and fulvic substances can participate in oxidation-reduction reactions via their quinone-like sites. They even hypothesized that there may be abiotic electron transfer involving complexed Fe3+ as a mediator. It can be hypothesized that FA participated in the degradation of CuO-NPs via oxidation-reduction reactions.

### 3.2. Characterization of CuO-NPs in Surface Water

CuO-NPs in surface water tended to remain in suspension/solution, where the amount of added Cu had a recovery rate close to 100%: 50% were found in the dissolved/small colloid fraction and the other 50% were associated with the nanometric form. CuO-NPs were distributed in all five of the defined size classes and were mostly present in the 55–76 and 77–137 nm classes. The surface water matrix tended to degrade CuO-NPs faster than MQ water. The same solution analyzed the next day (i.e., after 27 h) showed a recovery rate of the initial total Cu of almost 100%. On the other hand, the proportion of Cu associated with the dissolved fraction/small colloids was almost 80%, an increase of 30%. This increase was accompanied by a decrease in the proportion of nanoparticulate Cu, CuO-NPs, which was only 19%. This decrease resulted in a strong loss of agglomerate particles in the classes that included particles larger than 55 nm. However, there was an increase in particle numbers in the 48–55 nm class, which likely corresponded to degradation products of the largest particles. As in the case of pure MQ water, CuO-NPs degraded over time under these conditions, but to the largest extent.

CuO-NPs in surface water that was enriched in fulvic acid (FA) showed results relatively similar to those obtained with the solution prepared with surface water only. Indeed, nearly 90% recovery of Cu in FA-enriched water compared to fully recovered Cu in surface water was observed. Cu in the former solution was distributed among the dissolved/small colloids and nanometric fractions with proportions of 37% and 44%, respectively. In the surface water without FA added and analyzed after 3 h, the Cu partitioned almost equally between the dissolved/small colloid and nanometric fractions. The distribution of particles among the five size classes was similar. After 27 h, Cu recovery was 93%, with Cu distribution between the dissolved/small colloid and nanoscale fractions of 64% and 29%, respectively. The increase, compared to the analysis after 3 h, in the proportion of Cu in the dissolved fraction/small colloids, and the decrease in the nanoparticulate fraction, indicated that there was degradation of CuO-NPs within 27 h. The degradation of CuO-NPs was also observed with a decrease in particle detection numbers for size classes greater than 55 nm, while detection of the 48–55 nm class remained constant.

The surface water used for these experiments contained a natural organic carbon concentration of nearly 3 mg/L. Despite the low presence of FA in the solution of CuO-NPs in surface water, the role of the natural organic carbon in solution as a mediator for the degradation of CuO-NPs cannot be excluded, especially since this solution naturally contains Fe as well [22]. The solution prepared with surface water enriched with FA degraded CuO-NPs slightly more than the natural water that did not contain FA addition.

Overall, Figure 4 showed the highest recovery (near 100%) of Cu in solution and dispersion in surface water (SW) compared to pure MQ water. The effect of added FA decreased the Cu remaining in SW, mainly the undegraded nanoparticulate fraction. In contrast, the dissolved Cu plus small colloids (D + SC) fraction was more significant in MQ water with added FA, suggesting high NP degradation.

In conclusion, to fully understand the environmental impacts of CuO-NPs, our study showed the importance of investigating the physical and chemical forms of CuO-NPs over time and in different aqueous matrices as they transform. In evaluations of their bioaccumulation in aquatic organisms, these particles must be characterized to properly identify the state of the nanoparticle that defines what metal forms aquatic organisms are exposed to. Despite that one can consider CuO-NPs as not extremely toxic (e.g., effective concentrations ~500 ug/L [10], and even a much higher LC50 of >100 mg/L of green synthesized CuO-NPs [23]), their transformation products under environmental conditions may represent a higher exposure risk [11]. The effects and bioavailability of the most prevalent nanoforms and transformation products in different types of waters should be further investigated and considered in environmental risk assessments.

## Data Availability

All datasets analyzed or generated during the study are provided in this paper.
